# Inhibition Effects of Infrared Radiation Prior to Cold Storage Against *Alternaria alternata* on Yellow Peach (*Amygdalus persica*)

**DOI:** 10.3390/toxins17030106

**Published:** 2025-02-26

**Authors:** Longxiao Liu, Kai Fan, Qingwen Huang, Xinyi Wang, Dongxia Nie, Zheng Han, Zhizhong Li, Zhihui Zhao

**Affiliations:** 1 School of Life Science and Engineering, Lanzhou University of Technology, 287 Langongping Road, Lanzhou 730050, China; liulongxiao2025@163.com; 2 Institute for Agro-Food Standards and Testing Technology, Shanghai Academy of Agricultural Sciences, 1000 Jingqi Road, Shanghai 201403, China; fankai@saas.sh.cn (K.F.); huangqingwen@saas.sh.cn (Q.H.); wangxinyi_2020@163.com (X.W.); niedongxia@saas.sh.cn (D.N.); hanzheng@saas.sh.cn (Z.H.)

**Keywords:** *Alternaria alternata*, *Alternaria* toxins, infrared radiation, yellow peach, inhibitory effect

## Abstract

During postharvest storage, the yellow peach (*Amygdalus persica*) is susceptible to infection by *Alternaria alternata*, which causes fruit decay and produces multiple *Alternaria* toxins (ATs), leading to economic losses and potential health risks. The present study investigated the inhibitory effects of infrared radiation treatment against *A. alternata* on yellow peaches. Our in vitro experimental results indicated that infrared radiation at 50 °C for 30 min could completely inhibit fungal growth and AT production. Furthermore, infrared treatments prior to cold storage effectively delayed the onset of decay and significantly reduced the lesion diameter, decay rate, and AT levels in the yellow peaches inoculated with *A. alternata*. After the peaches underwent infrared radiation at 50 °C for 30 min and then cold storage for 60 days, the levels of tenuazonic acid, alternariol, alternariol methyl ether, and altenuene in the yellow peaches decreased by 95.1%, 98.6%, 76.1%, and 100.0%, respectively. Additionally, infrared radiation caused slight changes in their firmness, total soluble solids, and concentrations of sugar and organic acids, indicating minor negative impacts on the quality of the yellow peaches. Therefore, the present work provides a novel strategy for controlling *A. alternata* and AT contamination, thereby extending the shelf-life of yellow peaches, and improving food safety administration.

## 1. Introduction

China, the largest producer of peaches (*Prunus persica* L. Batsch), which are the sixth most important fruit globally, accounts for 54% of the world’s harvested area and 64% of the total yield [1]. Yellow peach (*Amygdalus persica*), one of the main categories of peach, is favored by consumers due to its unique organoleptic properties and rich nutrient compositions, including abundant vitamins, phenolic compounds, carotenoids, and trace elements [2]. However, yellow peaches are highly susceptible to fungal contamination due to their high moisture content, high level of nutrients, and thin skin. Additionally, the harvesting season for yellow peaches falls within the warmer months, and they experience vigorous respiration during postharvest storage. Thus, yellow peaches are prone to decay and spoilage, resulting in a short storage time and limited market circulation [3]. In China, the loss of yellow peaches after harvesting can escalate to approximately 20~30% [4].

*Alternaria* species, a prevalent genus encompassing pathogenic, endophytic, and saprophytic fungi, are known to cause spoilage in various fruits and vegetables during their postharvest shelf-life, such as apples, pears, grapes, tomatoes, and peaches [5,6,7,8]. Additionally, *Alternaria* species produce approximately 70 toxic secondary metabolites, termed *Alternaria* toxins (ATs), including tenuazonic acid (TeA), alternariol (AOH), alternariol methyl ether (AME), tentoxin (TEN), and altenuene (ALT), which have been frequently detected in a variety of fruits and vegetables, including tomatoes, potatoes, peaches, strawberries, pears, and apples [5,9,10,11,12,13,14,15]. It has been reported that ATs can lead to teratogenic, mutagenic, carcinogenic, and cytotoxic effects, thereby posing a serious health risk to humans and animals [16,17,18,19]. Consequently, it is imperative to utilize efficient storage and preservation strategies to inactivate *Alternaria* and reduce AT production, thereby extending the shelf-life of yellow peaches.

Low-temperature storage is the predominant approach to prolonging the shelf-life of fruits, as it can decrease the metabolic rate, delay senescence, reduce decay, and maintain the quality of fruits [20,21]. However, *Alternaria* species exhibit remarkable resilience, being capable of thriving and multiplying in environments with low temperatures and high humidity, causing deterioration and spoilage in fruits and vegetables stored in cold conditions [22,23]. In particular, our previous study found that high contents of ATs were produced in yellow peaches at 4 °C even though the fungal growth of *Alternaria alternata* (*A. alternata*) was suppressed [24]. Thus, it is necessary to incorporate cold storage with additional sterilization techniques to suppress *Alternaria* infection and AT production further in yellow peaches.

Currently, the main sterilization methods employed to ensure the quality and safety of fruits and vegetables are chemical treatments like washing, chemical surface cleaning, and the application of synthesized fungicides [25]. Nevertheless, ordinary water washing has a rather limited effect on removing microorganisms, while surface cleaning with chemical agents (sodium carbonate, chlorinated water, ozonated water, peroxide, etc.) may not only have a negative impact on the nutritional quality of the fruits and vegetables but also affect human health and the environment adversely [26,27,28]. Synthesized fungicides can effectively control microorganisms in postharvest fruits and vegetables. However, the issue of excessive residue has always been a major concern for consumers [29]. Moreover, the emergence of resistant fungal strains makes it difficult to develop a long-term efficient control strategy [30]. Compared to conventional chemical methods, various physical approaches, including gamma rays, X-rays, energetic electron beams, and ultraviolet light, possess the advantages of high effectiveness, energy efficiency, and the absence of residue, and their use for the decontamination of diverse food materials has been reported [31,32]. Infrared (IR) radiation has likewise been employed as a nascent non-chemical method for food decontamination. IR constitutes a segment of the non-ionizing electromagnetic spectrum, bridging the visible spectrum and microwaves, with a wavelength ranging from 0.75 to 1000 μm [33]. Due to its characteristics of high thermal efficiency, a fast heating rate, a rapid response time, and low energy consumption, IR has been widely applied and used in many food manufacturing processes, such as blanching, drying, thawing, roasting, baking, and cooking [34,35].

Recently, the application of IR to microbial inactivation has gained increasing interest. IR treatment inactivates microorganisms by causing thermal damage to their intracellular components, the cell envelope, and/or cell proteins and by leading to cytoplasm condensation and cellular content leakage [36]. Research has reported that microbial inactivation using IR is more effective than convection heating in both bacterial spores and vegetative cells [37], which is attributed to the efficient heat transfer by radiation and the high energy absorption of the microorganisms. However, IR radiation has a limited penetration capacity, rendering it particularly suitable for surface decontamination [34]. At present, IR decontamination technology has started to be commercially applied. For instance, a sequential IR and hot air roasting (SIRHA) approach for almonds, pistachios, and walnuts has been developed. This approach can shorten the roasting time and greatly reduce the levels of microbes such as *Salmonella Enteriditis* and *Enterococcus faecium* [38,39]. Furthermore, a commercial SIRHA system for drying walnuts resulted in drying time savings of 14% to 27% and energy savings of 10% to 20% with an improved quality and safety of the walnuts [35].

With regard to fungal decontamination, IR can be considered an emerging technique, and its applications to various foods, including corn, rice, peanuts, shiitake mushrooms, and so on [40], have been reported. For instance, IR treatment of the surface of mung beans at up to 70 °C for 5 min led to the complete inactivation of fungal growth of *Aspergillus flavus* and *Aspergillus niger* [41]. Similarly, heating shelled corn grains with an IR intensity of 3.24 kW·m^−2^ for 15 s resulted in complete fungal inactivation of *Aspergillus flavus* [42].

Although previous research has demonstrated the efficacy of IR for fungal decontamination, these studies have mainly focused on cereals or low-moisture foods. Few studies have been dedicated to fruits. Moreover, the effects of IR treatment on the growth of *A. alternata* and the production of ATs have hitherto not been reported. Therefore, for the first time, this study aimed to explore the effects of IR treatment prior to cold storage on *A. alternata* growth and AT production in yellow peaches. Additionally, the impact of the IR treatment on the quality of the yellow peaches was also evaluated.

## 2. Results and Discussion

### 2.1. The Inhibitory Effects of Infrared Treatments Against A. alternata on PDA Medium

**Figure 1:**
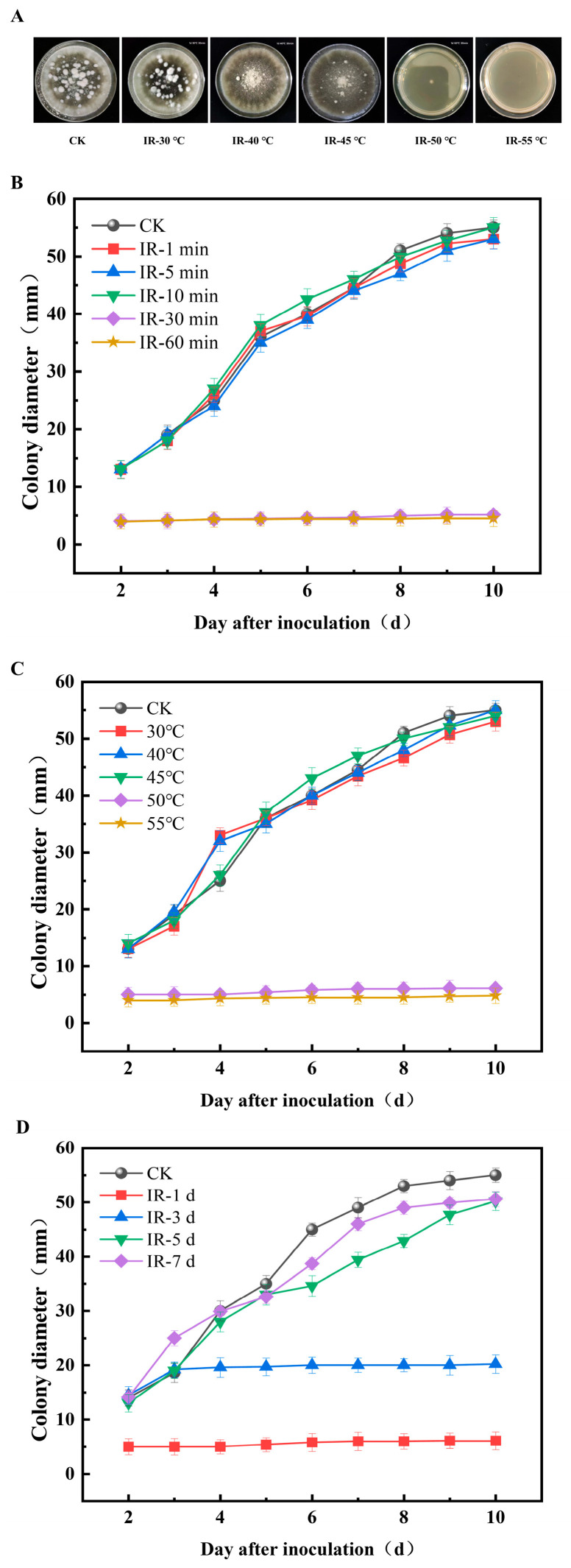
Inhibitory effects of infrared (IR) treatments on the colony morphology of *Alternaria alternata* on PDA medium (**A**). Inhibitory effects of infrared treatments on the colony diameter of *A. alternata* with different heating temperatures (**B**), durations (**C**), and applied periods (**D**).

To investigate the inhibitory effects of infrared treatment in vitro, the mycelial growth of *A. alternata* on PDA medium was evaluated. As shown in Figure 1A,B, under the IR treatment at a temperature below 50 °C, despite obvious variations in the colony morphology, no significant difference in the colony diameter of *A. alternata* was observed between the treatment group and the control (CK) group. When the IR temperature was increased to 50 °C and 55 °C, the growth of the strain was completely inhibited under the IR treatment. In contrast, the group treated at a sole heating temperature of 50 °C (T-50 °C) showed no inhibitory effect, indicating that the IR treatment rather than the heat treatment played the dominant role in the inhibition of *A. alternata* (Figure S1A). This was consistent with the results of a previous study which reported the inactivation effects of IR treatment on *Penicillium sp.* and *Cladosporium sp*. [37]. This was because the IR treatment could destroy the internal components within the fungal cells, including cell envelopes, proteins, DNA, RNA, and ribosomes, thereby restraining fungal development [33,40,43].

Besides the heating temperature, duration also significantly influenced the efficacy of IR decontamination [36]. As presented in Figure 1C, compared to the CK group, the IR treatments with durations of 1, 5, and 10 min exhibited no effect on the growth of *A. alternata*, whereas the treatment with a duration exceeding 30 min led to its complete inhibition. Therefore, a prolonged duration of IR treatment could significantly enhance the inhibitory effects against *A. alternata*, which was generally consistent with the results reported for IR treatment against *Pediococcus* spp. on almonds and *Aspergillus niger* on shiitake mushrooms [44,45]. This was attributed to the fact that these IR treatments could rapidly increase the surface temperature of the food and prolong the exposure of the microorganisms to heat stress, preventing cell repair after brief exposure to high temperatures. Furthermore, the effects of IR treatment against *A. alternata* at various time points after inoculation were also evaluated. The results showed that the growth of *A. alternata* was effectively inhibited when it underwent IR treatment on the first and third days after inoculation (Figure 1D). Nevertheless, the IR treatment applied more than three days after inoculation did not exhibit an inhibitory effect. Therefore, it is important to perform IR treatment during the initial stage of strain occurrence for the decontamination of *A. alternata*.

**Figure 2:**
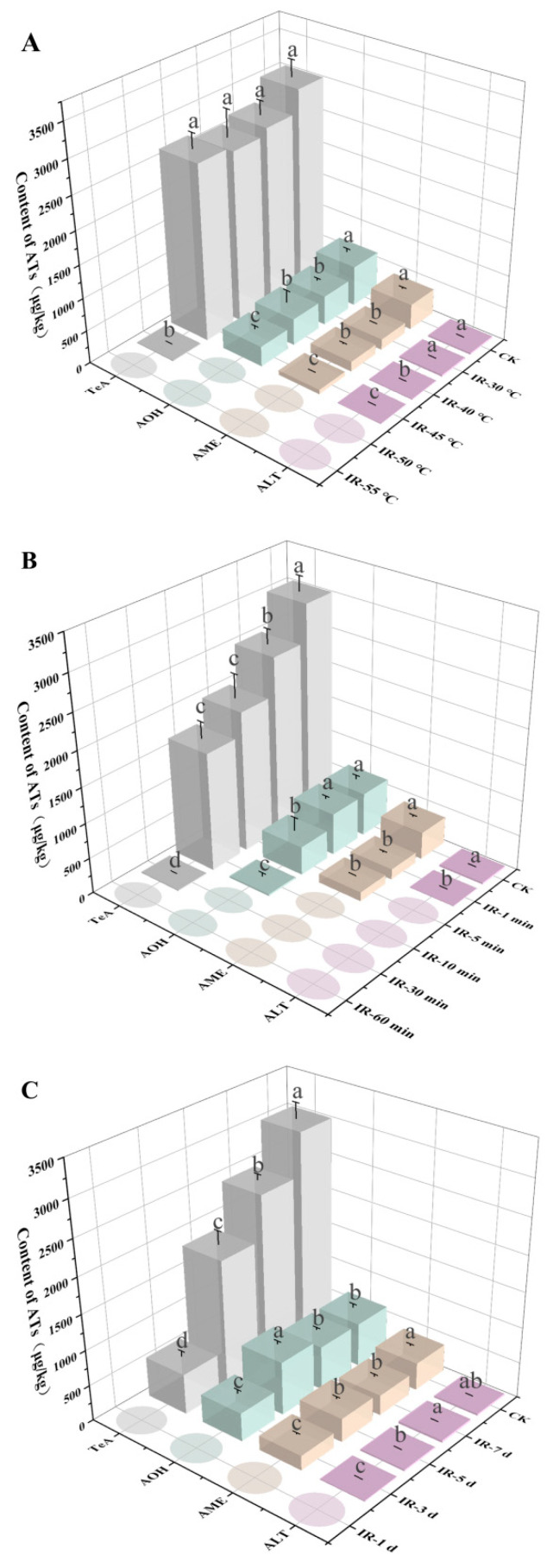
Inhibitory effect of infrared (IR) treatments on the production of *Alternaria* toxins (ATs) in PDA medium with different heating temperatures (**A**), durations (**B**), and applied periods (**C**). TeA = tenuazonic acid; AOH = alternariol; AME = alternariol methyl ether; and ALT = altenuene. Different lowercase letters indicate significant differences (*p* < 0.05). Circles indicate that the AT level was lower than the limit of quantification (LOQ).

In addition to inhibiting strain growth, IR treatment could also suppress the AT production. In comparison to the CK group, in the treatment group with IR treatment at 50 °C, the levels of the production of TeA, AME, AOH, and ALT by *A. alternata* MY-41 were significantly reduced, by 99.4%, 97.1%, 92.1%, and 100.0%, respectively, while no significant decrease was observed in the sole heating group (T-50 °C) (Figure S1B). When the IR temperatures were below 50 °C, there was no significant decrease in the levels of the ATs, consistent with the effects of the IR temperatures on strain growth (Figure 2A). Similarly, the IR treatments with durations of 30 and 60 min completely inhibited AT production (Figure 2B). However, the AT levels were also significantly reduced in the IR treatments with durations of 1, 5, and 10 min, even though the strain growth was not affected. Thus, the reduction in AT levels might not be solely attributed to the inhibition of fungal growth but could also be due to damage to the DNA associated with AT biosynthesis caused by the IR treatments [36], which requires further research to verify. Moreover, the lowest AT contents were also detected in the PDA plates that underwent IR treatment on the first day after inoculation (Figure 2C), suggesting the significance of the application of IR treatment during the early postharvest stage to ensure consumer safety [46].

### 2.2. Inhibitory Effects of the Infrared Treatments on the Growth of A. alternata on Yellow Peaches

**Figure 3:**
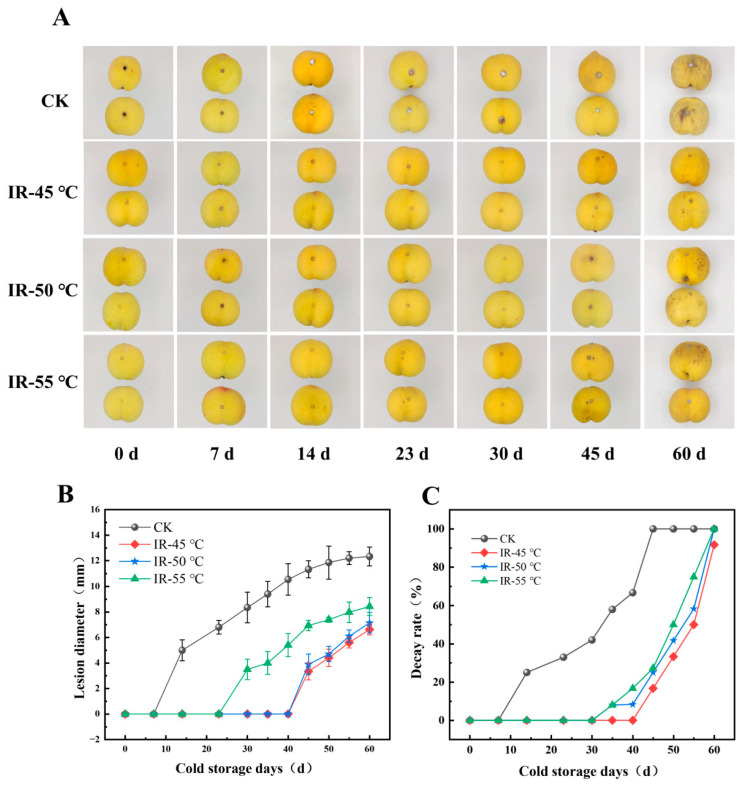
Effects of different infrared (IR) treatments on the appearance alterations (**A**), lesion diameter (**B**), and decay rate (**C**) in the yellow peaches inoculated with *A. alternata* kept at 4 °C and 70% relative humidity for 60 days.

Based on the preliminary experimental results from the PDA medium, yellow peaches artificially inoculated with *A. alternata* were immediately subjected to IR treatments at three temperatures (45 °C, 50 °C, and 55 °C) for 30 min. After IR treatment, no obvious changes were observed in the fruit’s size, shape, or color in the yellow peaches (Figure 3), which are important indicators for determining their commercial quality grade. Then, the inhibitory effects of the IR treatments on the growth and AT production of *A. alternata* in peaches kept at 4 °C for 60 days were evaluated. As shown in Figure 3A, on the 14th d of cold storage, a remarkable amount of white mycelium was observed on the yellow peaches in the CK group, indicating that their commercial value had declined. This was because *A. alternata* could be activated upon contact with wounds on the yellow peaches. Subsequently, germ tubes began to sprout and secrete cell-wall-degrading enzymes, which penetrated the cell wall and infected the host cells, from which they absorbed nutrients and established a parasitic relationship with the host [47]. However, compared to that in the CK group, the onset of rotting in the yellow peaches was delayed in the IR treatment group. Towards the end of the 60-day storage period, some mycelium was observed on the yellow peaches that underwent IR treatment at 55 °C (IR-55 °C). In contrast, no noticeable mycelium was observed on the yellow peaches that underwent IR treatment at 45 °C (IR-45 °C) and 50 °C (IR-50 °C) throughout the entire trial. Nevertheless, by the 60th d of cold storage, all of the yellow peach samples exhibited blackened skin, dehydration, and shriveling, rendering them devoid of commercial or edible value.

As illustrated in Figure 3B, the CK group experienced a rapid increase in the disease spot diameter after 14 days. In contrast, the diameter of the disease spots in the IR treatment groups exhibited a slight increase during the later period of storage while remaining significantly lower than that in the CK group. The decay rates of the yellow peaches during cold storage are shown in Figure 3C. In the CK group, the peaches began to decay at 14 d, with the decay rate reaching 42% at 30 d and the fruit being completely rotten at 45 days. Whereas decay started in the IR-50 °C and IR-55 °C groups at 35 d and the fruit became completely rotten at 60 d, meanwhile, the fruit in the IR-45 °C group started to decay at 40 d, with a decay rate of 91.7% at 60 d. Therefore, the IR treatment implemented prior to cold storage could effectively suppress the infection of the yellow peaches by *A. alternata* and reduce the decay rate of the fruit, thereby extending their storage period and ensuring their commercial value.

### 2.3. The Inhibitory Effects of Infrared Treatments on the Content of ATs on the Yellow Peaches

**Figure 4:**
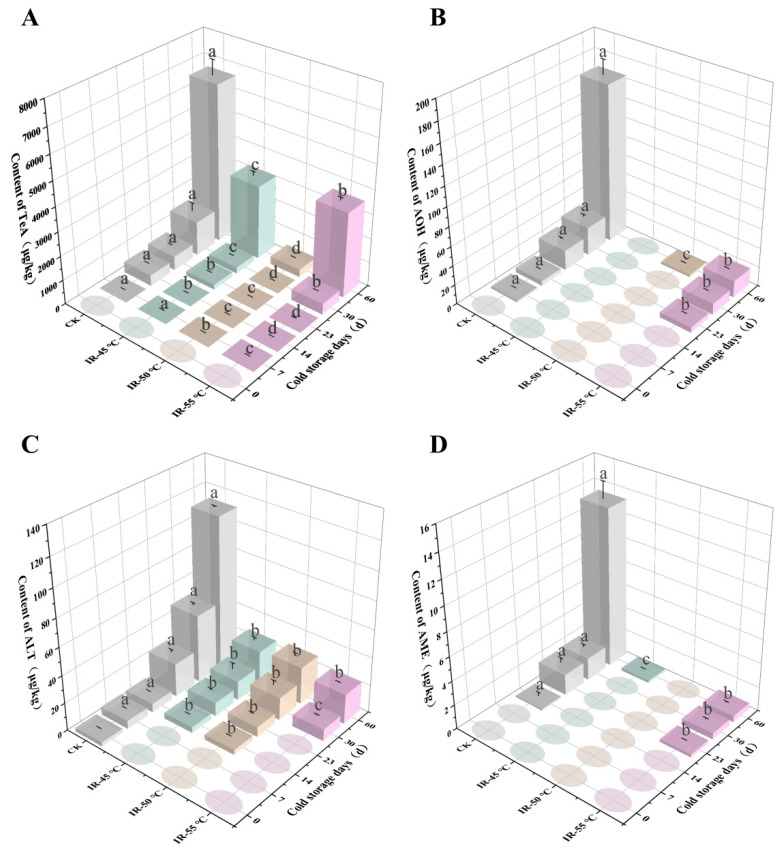
Effects of different infrared (IR) treatments on the contents of (**A**) tenuazonic acid (TeA), (**B**) alternariol (AOH), (**C**) altenuene (ALT), and (**D**) alternariol methyl ether (AME) in yellow peaches inoculated with *A. alternata* kept at 4 °C and 70% relative humidity for 60 days. Different lowercase letters within samples at the specified time indicate significant differences (*p* < 0.05). Circles indicate that the *Alternaria* toxin level was lower than the limit of quantification (LOQ).

Consistent with the results on the PDA medium, four ATs, namely TeA, AOH, ALT, and AME, were detected in the yellow peach samples (Figure 4). As the storage time was prolonged, the contents of ATs in all of the yellow peaches increased. The AT levels in the yellow peaches with IR treatment were significantly lower than those in the CK group (*p* < 0.05). In the IR treatment groups, higher contents of TeA and AOH were found in the yellow peaches that underwent IR treatment at 55 °C (IR-55 °C). It was speculated that the excessively high heating temperature might have enhanced the susceptibility of the yellow peaches to fungal infection, thereby resulting in higher levels of ATs and an increased rate of decay, as shown in Figure 2. In contrast, the greatest decrease in AT levels was observed in the IR treatment group treated at 50 °C (IR-50 °C), as the contents of TeA, AOH, ALT, and AME at 60 d were reduced by 95.1%, 98.6%, 76.1%, and 100.0%, respectively.

Although several previous studies have determined the effectiveness of IR treatment in the inactivation of fungi, only a limited amount of research has evaluated its effects on mycotoxin decontamination, mainly focusing on aflatoxins. Zavala-Franco et al. [48] reported that IR heating could reduce the contents of aflatoxins in tortillas by 71–73%. Ji et al. [49] reported that IR reduced more than 56% of the total amount of aflatoxin B1 content after treatment at 115 °C for 5 min. For Italian and Turkish hazelnuts, the residual aflatoxin levels were lower than 5% and 15%, respectively, after IR treatments at 140 °C for 40 min [50]. To our knowledge, the present study is the first to evaluate the effect of infrared on the control of *A. alternata* and ATs. Its results indicated that IR treatment could effectively suppress the AT production of *A. alternata*, thereby reducing the risk of mycotoxin contamination and spoilage in yellow peaches.

### 2.4. The Effects of the Infrared Treatments on the Morphology of the Spores and Mycelia of A. alternaria

**Figure 5:**
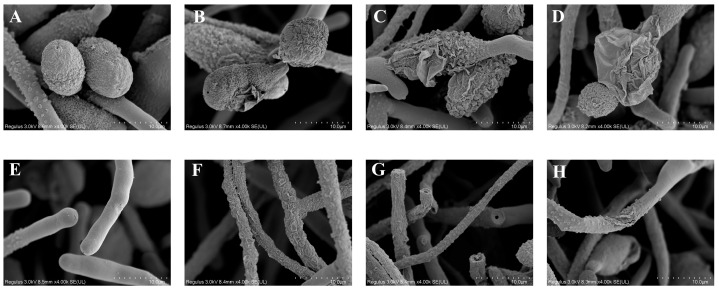
SEM images showing morphological alterations in untreated spores (**A**) and spores treated with infrared at 45 °C (**B**), 50 °C (**C**), and 55 °C (**D**), as well as untreated mycelia (**E**) and mycelia treated with infrared at 45 °C (**F**), 50 °C (**G**), and 55 °C (**H**).

SEM images were used to observe the morphological alterations in the fungal spores and the mycelia of *A. alternaria* that underwent the IR treatments. As shown in Figure 5A, the untreated spores of *A. alternaria* appeared to be oval, with a flat surface, a well-defined outline, and a full shape. After the IR treatment at 45 °C, the spores revealed a slight degree of wrinkling, and their outlines became irregular (Figure 5B). With an increase in the heating temperature (50 °C and 55 °C), the spores’ surfaces underwent shrinkage, more severe wrinkling, and damaged membrane integrity (Figure 5C,D), which could be attributed to the fact that IR sterilization mainly relies on a thermal effect and characteristic absorption [35]. Similarly, the mycelia in the CK group had a linear shape and a smooth surface without wrinkles, appearing plump and fully stretched (Figure 5E). Conversely, the mycelia that underwent IR treatment exhibited rough and shrunken surfaces and even displayed a ruptured and distorted appearance (Figure 5F–H). These results indicated that the IR treatment disrupted the morphology of the spores and mycelia, thereby inhibiting the normal growth of *A. alternata.* Similar results were observed in reports of IR treatment of *Aspergillus flavus* and *Aspergillus niger* [41,51]. It was documented that the inactivation of fungal spores treated with IR was due to transformations in the coats of the spores and physical destruction of the cells.

### 2.5. The Effects of Infrared Treatment on Fruit Quality in the Yellow Peaches

The application of IR treatment depends on attaining the optimal trade-off that achieves inhibitory effects on fungal growth and mycotoxin production while ensuring food quality [52]. Firmness and total soluble solids (TSSs) are representative indicators reflecting the quality of yellow peaches [3]. As shown in Table 1, after cold storage, the skin’s firmness declined in both the control and the IR treatment group. However, the flesh’s firmness remained substantially stable, which was in line with the results reported for ’Fuli’ peaches and ‘Early Rich’ peaches stored at a low temperature [53,54]. These results can be explained as symptoms of chilling injuries (CIs), which cause uneven ripening and dry textures in fruits [55]. Compared with the control group, the groups with the IR treatments exhibited no significant changes in skin and flesh firmness at the initial stage of storage. However, significant differences in the skin and flesh firmness values were observed as the storage time was extended. Regarding TSSs, the values in all of the yellow peaches ranged from 9.99 ± 0.83% to 11.53 ± 1.04% (Table 1), which suggested that they could be classified as first-grade according to the China Agricultural Industry Standards (NY/T 586-2002) [56]. There was no obvious difference between the IR treatment group and the CK group on the same day, indicating that the TSSs (equivalent to sweetness) were not affected by the IR treatment. For all of the yellow peach samples, the TSS value peaked on the 23rd d and then exhibited a rapid decline, which was generally consistent with the observations for peaches irradiated with ultraviolet reported by Zhou et al. [57]. Thus, these results indicated that the IR treatment had no significant effect on the physiological properties of the yellow peaches, but there was a potential slight impact after their long-term storage.

**Figure 6:**
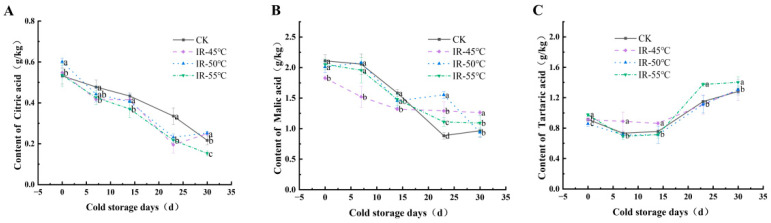
Contents of citric acid (**A**), malic acid (**B**), and tartaric acid (**C**) in yellow peaches stored at 4 °C and 70% relative humidity for 30 days. Different letters within samples at the specified time indicate significant differences (*p* < 0.05).

As a principal quality trait influencing consumer acceptance, fruit’s flavor attributes are heavily influenced by its compositions and concentrations of sugars and acids [57]. The predominant sugars in the yellow peaches were sucrose, fructose, and glucose, whereas citric acid, malic acid, and tartaric acid comprised the major organic acids. As shown in Figure 6, the contents of citric acid and malic acid in all of the yellow peaches decreased as the storage time was prolonged, while the tartaric acid content initially declined and then increased during storage. Compared with that in the control group, the citric acid content in the IR-50 °C group was significantly lower during the 7−23 d period of storage but was higher at 30 d (Figure 6A). Regarding malic acid and tartaric acid (Figure 6B,C), there was no significant difference between these values in the IR-50 °C group and the control group for the majority of the storage time, except that the malic acid content in the IR-50 °C group was significantly higher at 23 d. Consequently, the IR treatment at 50 °C exerted a marginal effect on the organic acid content of the yellow peaches, with the change remaining within an acceptable range.

**Figure 7:**
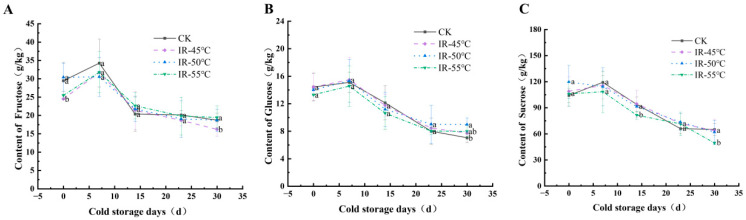
Contents of fructose (**A**), glucose (**B**), and sucrose (**C**) in yellow peaches stored at 4 °C and 70% relative humidity for 30 days. Different letters within samples at the specified time indicate significant differences (*p* < 0.05).

Moreover, the contents of fructose, glucose, and sucrose in the yellow peaches in each group demonstrated an overall trend of an initial increase followed by a decrease during storage (Figure 7). No significant difference was observed in the sugar contents between the IR-50 °C group and the control group in most cases, indicating that IR treatment at 50 °C had no impact on the sweetness of the yellow peaches. Notably, the yellow peaches in the IR-50 °C group exhibited a higher glucose concentration at 30 d (Figure 7B). Therefore, 50 °C was determined to be the optimum temperature for achieving control over *A. alternata* while simultaneously obtaining high-quality yellow peaches.

## 3. Conclusions

The present study demonstrated the implementation of IR treatment prior to cold storage for the effective control of *A. alternata* and ATs in yellow peaches for the first time. It could disrupt the morphology of the spores and mycelia, inhibit *A. alternata’s* growth and AT production, and significantly reduce the lesion diameters and decay rates in yellow peaches during cold storage. On the other hand, the IR treatment imposed minor negative impacts on the firmness, TSSs, and concentrations of sugar and organic acids in the yellow peaches. Hence, this study demonstrated the potential of IR treatment to ensure the quality and safety of yellow peaches. However, certain important thermolabile nutrients, such as vitamins, whose content could be strongly related to the temperature reached inside the fruit, were not considered in the present study. Moreover, the long-term impacts of IR on the sensory and biochemical characteristics of the yellow peaches were not investigated either, which is another limitation of the present study. Therefore, further studies employing more advanced analytical techniques are required to comprehensively evaluate the effects of IR treatment on the physicochemical properties, sensory attributes, and nutritional values of fruits and vegetables and the mechanisms of these effects to facilitate its large-scale practical application.

## 4. Materials and Methods

### 4.1. Reagents and Chemicals

Acetonitrile and methanol (HPLC-grade) were purchased from Merck (Darmstadt, Germany). Sodium chloride (NaCl, analytical-grade), anhydrous magnesium sulfate (MgSO4, analytical-grade), and ammonium acetate (HPLC-grade) were purchased from ANPEL (Shanghai, China). Potato dextrose agar (PDA), octadecylsilane-bonded silica gel (C_18_), and propylethylenediamine (PSA) were purchased from Wuzhi Biotechnology (Shanghai, China). The analytical standards (stock solutions) of AOH (100.0 µg/mL), AME (100.3 µg/mL), ALT (101.1 µg/mL), and TeA (101.1 µg/mL) dissolved in acetonitrile were obtained from Romer Labs (Union, MO, USA). The analytical standard powders of citric acid (25 mg), malic acid (100 mg), tartaric acid (250 mg), frutose (250 mg), glucose (250 mg), and sucrose (200 mg) were purchased from ANPEL (Shanghai, China). The fixative for SEM (2.5% glutaraldehyde solution) was purchased from Solarbio Science & Technology (Beijing, China).

### 4.2. Fruit Materials and Fungal Strains

Fresh yellow peaches (‘Jinxiang’) at a commercially mature stage (approximately 80 d post-anthesis, exhibiting 80–90% yellow peel coloration) were harvested from orchards located in Fengxian District, Shanghai, China. The harvested yellow peaches were placed into foam boxes and then transported to the laboratory at the Institute for Agro-food Standards and Testing Technology, Shanghai Academy of Agricultural Sciences (Shanghai, China), within 1 h for experimental processing. A total of 560 yellow peaches, uniform in size (about 250–300 g, with an average weight of 281.02 ± 41.56 g) and free from physical defects or apparent infections, were selected and randomly divided into two groups. The first group received artificial inoculation of *A. alternata* and subsequent IR treatment. The second group was directly treated with IR radiation without artificial inoculation, followed by a quality evaluation.

The strain of *A. alternata* (MY-41), which is capable of producing four typical ATs (TeA, AOH, AME, and ALT), was isolated from a yellow peach through single-spore isolation and preserved at the Institute for Agro-food Standards and Testing Technology, the Shanghai Academy of Agricultural Sciences. Prior to the experiments, *A. alternata* was sub-cultured on the PDA medium at 28 °C for 10 days. Then, the conidia were collected using a cotton swab within sterile water, and the mycelia were removed through filtration with four layers of sterile gauze. The resulting spore suspension was then adjusted to a concentration of 2 × 10^6^ conidia/mL with sterile water and prepared for inoculation.

### 4.3. Infrared Heating Processing

A custom-designed IR heating device (Tiancheng Experimental instrument Manufacturing Co., Ltd., Shanghai, China) was equipped with an IR generator with a length of 50 cm (Meibo Infrared Technology Co., Ltd., Zhenjiang, China), a sample tray holder with an adjustable distance, and a processing chamber with dimensions of 60 (length) × 40 (width) × 30 (height) cm. The heating unit generated infrared radiation at a wavelength of 2–5 μm with a power level of 276 W. The samples of yellow peaches or the PDA plates were placed 10 cm below the bottom edge of the wave guide.

### 4.4. The Inhibitory Effects of Infrared Treatments Against A. alternata

The in vitro inhibitory effects of the infrared treatment against *A. alternata* were evaluated on PDA medium. Under sterile conditions, 20 µL of the conidial suspension of *A. alternata* (2 × 10^6^ conidia/mL) was pipetted into the center of the pre-made PDA substrate plate, and it was then incubated at 28 °C in the dark for 10 d. On different days after incubation (1, 3, 5, and 7 d), the PDA plates were subjected to IR treatment to target temperatures of 50 °C for 30 min. Inoculated PDA plates without IR treatment were set as the control (CK). The colony diameters were measured using the crossing method every day. Specifically, the diameters were measured both horizontally and vertically, and then the average value was calculated. In each treatment and control group, there were ten biological replicates. After incubation, the AT concentrations were analyzed further using ultra-high-performance liquid chromatography–tandem mass spectrometry (UPLC-MS/MS).

### 4.5. The Inactivation Effect of the Infrared Treatment on A. alternata in Yellow Peaches

To investigate the in vivo inactivation effect of the IR treatment, the yellow peaches were artificially inoculated with *A. alternata*. The yellow peaches were initially rinsed with tap water and then immersed in 75% alcohol for 2 min. Subsequently, they were rinsed with sterile water and dried with sterile gauze. Under sterile conditions, a 5 mm diameter wound was created in the equatorial area of the yellow peaches using a sterilized puncher. Then, 20 μL of the spore suspension of *A. alternata* (2 × 10^6^ conidia/mL) was inserted into the wound using a sterilized micropipette, and fruits with sterile water inserted were used as the control [24].

Based on the in vitro results, the inoculated yellow peaches underwent IR treatment to target temperatures of 45 °C (IR-45 °C), 50 °C (IR-50 °C), and 55 °C (IR-55 °C) for 30 min, respectively. Inoculated yellow peaches without the IR treatment were set as the control (CK). Then, all of the yellow peaches were stored in dark at 4 ± 1 °C and 70% relative humidity for different durations (7, 14, 23, 30, 45, and 60 d). Each treatment group and the control group consisted of 10 peaches. During the cold storage period, the lesion diameters and the decay rates in the yellow peaches were assessed. The decay of the yellow peaches was determined through visual evaluation, with the growth of fungi on the peaches being regarded as decay [58]. The decay rate was calculated as the number of rotted yellow peaches divided by the total number of yellow peaches and expressed as a percentage (%). After storage, the yellow peach samples were further homogenized for analysis of the ATs using UPLC-MS/MS (Waters, Milford, MA, USA).

### 4.6. Mycotoxin Analysis

Determination of the AT concentrations was performed according to the method previously established in our laboratory [59,60]. For the PDA medium, the mycelia and the medium were ultrasonically extracted with acetonitrile containing 1% formic acid. After vortexing and centrifugation, the supernatant was diluted with acetonitrile/water containing 5 mmol/L of ammonium acetate (20:80, *v*/*v*), filtrated, and then analyzed using UPLC-MS/MS. For the yellow peaches, the homogenized samples were ultrasonically extracted with acetonitrile/water (80:20, *v*/*v*) containing 1% acetic acid. After salting out with magnesium sulfate and sodium chloride and then purification with C_18_ and PSA, the supernatant was dried under nitrogen gas, re-dissolved with water containing 5 mmol/L of ammonium acetate/acetonitrile (50:50, *v*/*v*), filtrated, and analyzed using UPLC-MS/MS.

The UPLC analysis was performed using a Waters ACQUITY ultra-high-performance LC system (Waters, Milford, MA, USA). Separation was achieved on an XBridge BEH C_18_ column (3.0 mm × 100 mm, 2.5 µm, Waters, Milford, MA, USA). The mobile phase consisted of methanol (A) and water containing 5 mmol/L of ammonium acetate (B). A linear gradient elution program was set as follows: 0~0.2 min, 30%A; 0.2~5 min, 30%A~90%A; 5~7 min, 90%A; 7~7.5 min, 90%A~30%A; 7.5~8.5 min, 30%A; and flow rate: 0.4 mL/min. The injection volume was 3 µL.

For the MS/MS analysis, a Waters TQS mass spectrometer system (Waters, Milford, MA, USA) was used in positive electrospray ionization mode (ESI^+^) with the following parameters: Interface voltage of the capillary: 2.5 kV; Source temperature: 150 °C; Desolvation temperature: 500 °C. The nebulizing gas and desolvation gas flow rates were 7.0 bar and 1000 L/h, respectively. Multiple reaction monitoring (MRM) mode was used for the quantification and confirmation of the ATs, with the parameters shown in Table S1.

### 4.7. Scanning Electron Microscopy (SEM) Observation

A minorly modified version of the method proposed by Basak and Guha [61] was adopted to observe the damage induced in the mycelia and spores of *A. alternata* by the IR treatment using SEM (SU8100, Hitachi, Ltd., Tokyo, Japan). The treated mycelia and spores were collected from the PDA medium, washed with PBS (pH = 7.4), and prefixed with 2.5% glutaraldehyde solution overnight at 4 °C. The fixed mycelia and spores were harvested and dried with a critical-point dryer (K850, Quorom Technologies Ltd., Laughton, UK), mounted onto SEM stubs using conductive carbon tape, and then sputtered with Pt for about 120 s in an ion-sputtering instrument (MC1000, Hitachi, Ltd., Tokyo, Japan). The SEM observation was performed under the following analytical conditions: EHT = 3.00 kV and WD = 8.4 mm.

### 4.8. Quality Evaluation

After the IR treatment, the yellow peaches without inoculation were stored in the dark at 4 ± 1 °C and under 70% relative humidity for different durations (7, 14, 23, and 30 d). Each treatment group and the control group consisted of 10 peaches. During the cold storage period, firmness, TSSs, and the concentrations of sugar and organic acids were investigated to evaluate the effects of the IR treatment on the quality of the yellow peaches. The peel firmness and flesh firmness were determined using a texture analyzer (M2610, LOTUN SCIENCE Co., Ltd., Xiamen, China), featuring a computer-controlled motorized device equipped with an 8 mm diameter cylindrical probe. The probe penetrated the fruit tissues to a depth of 10 mm at a test speed of 1 mm/s. All of the experiments were performed in the equatorial section of each fruit sample, and the results were expressed as g. The TSSs were determined using a handheld refractometer (WZS 20, INESA Physico-Optical Instrument Co., Ltd., Shanghai, China), and this value was expressed as a percentage (%). The contents of organic acids (citric acid, malic acid, and tartaric acid) and soluble sugars (sucrose, glucose, and fructose) were determined using the method in [57] with some modifications. Briefly, homogenized samples were extracted using anhydrous ethanol/0.4% metaphosphoric acid (80:20, *v*/*v*). Subsequently, the liquid supernatant was concentrated and redissolved in ultrapure water to determine the soluble sugar contents using a Waters ACQUITY Arc high-pressure liquid chromatography system (Waters, Milford, MA, USA) with an Ultimate XB-NH_2_ column (5 μm × 4.6 mm × 250 mm, Welch Materials, Baltimore, MD, USA) and a differential refraction detector. The column temperature was 80 °C, and 15 µL samples were injected. The mobile phase was water containing 70% acetonitrile, and the flow rate was 1 mL/min. The organic acids were detected using an Agilment 1200 liquid chromatography system (Agilent, Palo Alto, CA, USA) with an Ultimate AQ-C_18_ column (5 μm × 4.6 mm × 250 mm, Welch Materials, Baltimore, MD, USA) and an ultraviolet detector. The mobile phase was water containing 0.1% phosphoric acid. The flow rate was 1 mL/min, the injection volume was 20 µL, and the ultraviolet wavelength was 210 nm.

### 4.9. Statistical Analysis

The experimental data were expressed as the mean and standard deviation (SD) of at least three independent replicates. All of the statistical analysis procedures were carried out using the statistical software SPSS 20 (SPSS, Inc., Chicago, IL, USA). The statistical significance was determined through a two-tailed one-way analysis of variance (ANOVA). Values of *p* < 0.05 were considered statistically significant. Origin 2024b (OriginLab, Inc., Northampton, MA, USA) was utilized to generate the graphical representations.

## Figures and Tables

**Table 1:** Effect of infrared treatment on the quality of yellow peaches uninoculated with *Alternaria alternata* during cold storage (*n* = 10).

Days of Cold Storage	Treatment	Skin Firmness (g)	Flesh Firmness (g)	TSSs (%)
0	CK	443.64 ± 54.17 a	43.80 ± 5.87 a	11.19 ± 0.64 a
	IR-45 °C	434.59 ± 59.09 a	47.17 ± 14.79 a	10.56 ± 0.59 a
	IR-50 °C	420.95 ± 70.63 a	43.83 ± 7.45 a	10.60 ± 0.50 a
	IR-55 °C	476.47 ± 66.6 a	49.4 ± 8.51 a	10.78 ± 0.92 a
7	CK	344.46 ± 70.9 a	50.4 ± 10.82 a	10.87 ± 0.54 a
	IR-45 °C	367.53 ± 91.54 a	47.29 ± 7.37 a	10.41 ± 0.47 a
	IR-50 °C	334.04 ± 78.32 a	42.03 ± 3.58 a	10.68 ± 0.58 a
	IH-55 °C	413.70 ± 91.75 a	48.89 ± 13.71 a	10.48 ± 0.54 a
14	CK	415.66 ± 82.1 a	36.44 ± 5.96 b	9.99 ± 0.83 b
	IR-45 °C	474.94 ± 147.82 a	37.82 ± 6.41 b	10.16 ± 0.50 ab
	IR-50 °C	443.15 ± 18.3 a	50.3 ± 5.65 a	10.74 ± 0.71 a
	IR-55 °C	457.70 ± 121.81 a	48.13 ± 3.67 a	10.48 ± 0.54 ab
23	CK	305.65 ± 36.66 a	38.20 ± 6.49 a	11.53 ± 1.04 a
	IR-45 °C	245.03 ± 31.36 b	42.28 ± 4.63 a	11.06 ± 1.2 a
	IR-50 °C	275.13 ± 38.72 ab	44.66 ± 9.49 a	10.85 ± 0.79 a
	IR-55 °C	282.25 ± 38.5 a	44.80 ± 3.43 a	10.63 ± 0.64 a
30	CK	282.00 ± 39.84 a	41.79 ± 7.91 b	10.41 ± 1.10 a
	IR-45 °C	223.57 ± 45.33 b	46.48 ± 8.92 ab	10.20 ± 0.63 a
	IR-50 °C	271.20 ± 46.66 a	57.08 ± 15.11 a	10.85 ± 0.58 a
	IR-55 °C	219.55 ± 39.62 b	42.18 ± 12.37 b	10.29 ± 0.76 a

Different lowercase letters indicate significant differences (*p* < 0.05).

## Data Availability

The original contributions presented in this study are included in the article/supplementary material. Further inquiries can be directed to the corresponding author(s).

## Supplementary Materials

The following supporting information can be downloaded at https://www.mdpi.com/article/10.3390/toxins17030106/s1. Figure S1: Effect of infrared treatments on the colony morphology of *Alternaria alternata* (A) and *Alternaria* toxin production (B) in PDA medium; Table S1: MS/MS parameters for the determination of the ATs.
