# Supplementary material for: Inhibition Effects of Infrared Radiation Prior to Cold Storage Against Alternaria alternata on Yellow Peach (Amygdalus persica)
Source: Toxins (Basel). 2025 Feb 26;17(3):106. doi: 10.3390/toxins17030106 (PMC11946141; doi:10.3390/toxins17030106)
Supplement: Supplementary file 1 [file toxins-17-00106-s001.zip › toxins-3466601-supplementary.pdf]

toxins

MDPI

Article

Supplementary Materials: Inhibition Effects of Infrared Radiation Prior to Cold Storage Against *Alternaria alternata* on Yellow Peach (*Amygdalus persica*)

Longxiao Liu [1,2,†], Kai Fan [2,†], Qingwen Huang [2], Xinyi Wang [2], Dongxia Nie [2], Zheng Han [2], Zhizhong Li [1,*] and Zhihui Zhao [2,*]

[1] School of Life Science and Engineering, Lanzhou University of Technology, 287 Langongping Road, Lanzhou 730050, China; liulongxiao2025@163.com

[2] Institute for Agro-Food Standards and Testing Technology, Shanghai Academy of Agricultural Sciences, 1000 Jingqi Road, Shanghai 201403, China; fankai@saas.sh.cn (K.F.); huangqingwen@saas.sh.cn (Q.H.); wangxinyi_2020@163.com (X.W.); niedongxia@saas.sh.cn (D.N.); hanzheng@saas.sh.cn (Z.H.)

* Correspondence: zzli2004@lut.edu.cn (Z.L.); zhihuizhao@saas.sh.cn (Z.Z.)

† These authors contributed equally to this work

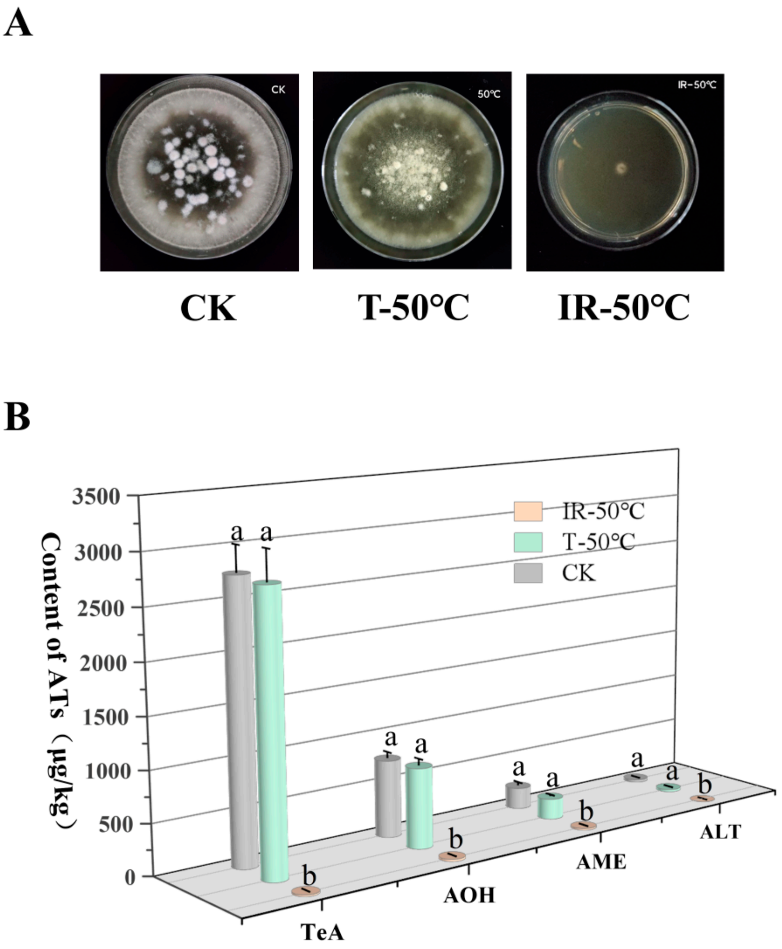

Figure S1. Effect of infrared treatments on the colony morphology of *Alternaria alternata (A)* and *Alternaria* toxins production (B) in PDA medium. CK=control without infrared treatment; T-50 °C = sole heating treatment at 50 °C; IR-50 °C=infrared treatment at 50 °C.Different lowercase letters indicated significant differences (*P* < 0.05).

Table S1. MS/MS parameters for the determination of the ATs.

**Alternaria Toxins**	**Precursor ion (*m*/*z*)**	**Product ions (*m*/*z*)**	**CEs (eV)**
TeA	198.1	125.0*, 153.1	12, 16
AOH	259.0	185.1*, 213.1	28, 24
AME	273.0	258.0*, 128.1	25, 26
ALT	293.0	257.1*, 275.1	14, 8

CEs: collision energies; *Quantitative ion.
